# Cannabidiol, a plant-derived compound, is an emerging strategy for treating cognitive impairments: comprehensive review of randomized trials

**DOI:** 10.3389/fphar.2024.1403147

**Published:** 2024-09-11

**Authors:** Adriana Yndart Arias, Kamila Vadell, Arti Vashist, Nagesh Kolishetti, Madepalli K. Lakshmana, Madhavan Nair, Juan P. Liuzzi

**Affiliations:** ^1^ Department of Dietetics and Nutrition, Robert Stempel College of Public Health and Social Work, Florida International University, Miami, FL, United States; ^2^ Department of Cellular and Molecular Medicine, Herbert Wertheim College of Medicine, Florida International University, Miami, FL, United States

**Keywords:** CBD, cannabidiol, cognition, clinical trial, drug of abuse, anxiety, Alzheimer’s disease, neurocognitive disorders

## Abstract

**Background:**

Finding new strategies to treat cognitive disorders is a challenging task. Medication must defeat the blood–brain barrier. Cannabidiol (CBD), a non-intoxicating compound of the cannabis plant, has gained recognition as a nutraceutical for its potential effectiveness in treating anxiety, oxidative stress, convulsions, and inflammation. However, the dose, tolerable upper intake, formulation, administration routes, comorbidities, diet, and demographic factors to reverse cognitive impairments have not been completely explored. Trials using CBD as a primary intervention have been conducted to alleviate cognitive issues. This review evaluates the benefits of CBD supplementation, research design, formulations, and outcomes reported in randomized clinical trials.

**Methods:**

An evidence-based systematic literature review was conducted using PUBMED and the Florida International University Research Library resources. Fourteen randomized trials were selected for review, and their designs and outcomes were compared conceptually and in the form of resume tables.

**Results:**

CBD showed improvement in anxiety and cognitive impairments in 9 out of 16 analyzed trials. However, the variability could be justified due to the diversity of the trial designs, underpowered studies, assayed population, uncontrolled results for comorbidities, medications, severity of drug dependence, compliances, and adherences. Overall, oral single doses of 200 mg–1,500 mg or vaporized 13.75 mg of CBD were shown to be effective at treating anxiety and cognition with a good safety profile and no drug addiction behaviors. Conversely, results that did not have a significant effect on treating cognitive impairments can be explained by various factors such as THC or other abuse drugs masking effect, low dose, and unknown purity of CBD. Furthermore, CBD shows potential properties that can be tested in the future for Alzheimer’s disease.

**Conclusion:**

As medical cannabis becomes more accessible, it is essential to understand whether medication rich in CBD exerts a beneficial effect on cognitive disorders. Our study concludes that CBD is a promising candidate for treating neurocognitive disorders; however, more studies are required to define CBD as a therapeutic candidate for managing cognitive disorders.

## Graphical Abstract

**Figure:**
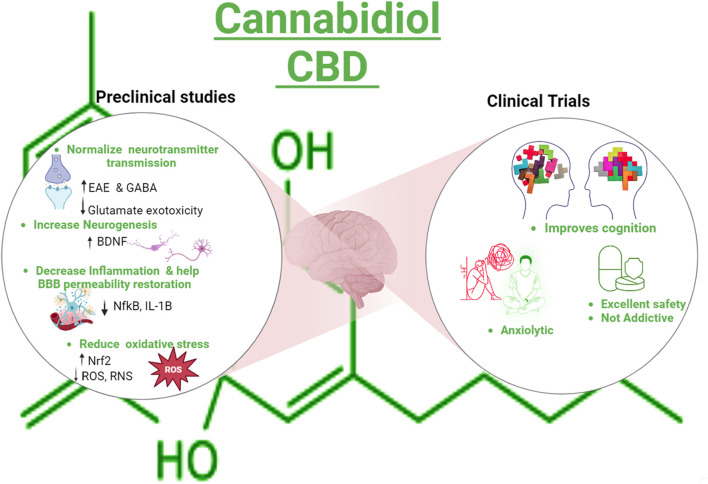
Created with Biorender.com.

## Introduction

Cannabidiol (CBD), a phytocannabinoid, is derived from the cannabis plant. Recently, CBD has gained significant attention due to its medical potential. It has anxiolytic, antioxidant, anti-inflammatory, antiemetic, and antipsychotic properties, making it a subject of interest in the scientific community (Mechoulam et al., 2002; Yndart Arias et al., 2023). In a recent review, we discussed the mechanism of action of CBD and its use in neurodegenerative diseases and preclinical studies related to literature (Bhunia et al., 2022). For instance, studies have shown that CBD enhances gamma-aminobutyric acid (GABA) synaptic transmission, a neurotransmitter associated with calming effects, and CBD consumption is related to the upregulation of the brain-derived neurotrophic factor (BDNF), indicating a potential to address anxiety-related conditions and cognitive issues (Augustin and Lovinger, 2022; Sales et al., 2019). In addition, preclinical evidence shows that CBD prevented the increase in blood–brain barrier (BBB) permeability in traumatic brain-injured mice, attenuating edema and neuroinflammation and helping recover neurological functions (Hampson et al., 1998; Prud’homme et al., 2015; Wang et al., 2022; Jiang et al., 2021). CBD counteracts the deficits of mRNA levels of AMPA receptor subunits (glutamate-gated ion channels), synaptophysin (SYP), DLG4, glial-cell-derived neurotrophic factor (GDNF), and BDNF in amyloid ß 1–42-induced mice (Chen et al., 2023). Similarly, TAU transgenic mice treated with CBD exhibited decreased anxiety and enhanced spatial reference memory impairment, which is partially explained by the fact that CBD is an agonist of the serotonin 1A receptor (5HT1A). Serotonin is a neurotransmitter depleted in depression and anxiety conditions (Chen et al., 2023). It has also been reported that CBD increases serotoninergic and glutamatergic transmission, modulating the serotonin receptor positively (Chen et al., 2023; Martinez Naya et al., 2023). Despite the promising properties, the Food Drug Administration (FDA) has not approved CBD as a food additive or supplement except for Epidiolex, a pharmaceutical and high-purity grade CBD oil used to treat epilepsy (Mechoulam et al., 2002; Karaźniewicz-Łada et al., 2021).

Of note, medications containing CBD can be administered through various routes, including oral, vaporized, intravenous, and intramuscular, and the administration route impacts the drug’s effectiveness. Absorption of orally administered CBD medications occurs in the small intestine, and its concentration diminishes before reaching systemic circulation (Kim and Jesus, 2023). In contrast, inhaled preparations bypass the small intestine and enter the systemic circulation, avoiding gastric degradation and requiring less CBD medication to obtain similar benefits. Therefore, the availability depends on the administration route: 11%–45% of the drug is available after inhaling, while 6% is available after the oral route (Chayasirisobhon, 2020). However, the entrance of drugs across the BBB (Kim and Jesus, 2023; Chayasirisobhon, 2020) depends on the lipid solubility, size, and charge of the substance. For example, CBD has a high lipophilicity grade, favoring the entrance to the brain (Chayasirisobhon, 2020). Each administration route has advantages and disadvantages, and the ideal route depends on the treatment’s specific target and end goals. In addition, CBD is hydroxylated by cytochrome P450 enzymes (CYP3A4 and CYP2C9) in the liver. Its plasma half-life varies from 18 h to 32 h, and it is excreted primarily in feces (Chayasirisobhon, 2020).

Cognition encompasses general mental processes critical to human functioning that include memory, attention, motor skills, language, and/or executive functioning (Bayne et al., 2019). Several neuropsychiatric conditions, including brain damage, stroke, degenerative dementia, amnestic populations, illnesses, experiences, trauma, or congenital abnormalities, can contribute to cognitive impairment (Bayne et al., 2019; Robbins, 2011). Moreover, anxiety can be closely intertwined with cognitive functioning. This primary emotional or primary response can significantly impact cognitive processes such as attention, memory, decision making, and problem solving (Hartley and Phelps, 2012; Alvi et al., 2022).

Because randomized controlled trials (RCT) are the most suitable method of answering questions about treatment (AND, 2022), we aimed to provide a comprehensive overview of clinical trial results examining the effects of CBD on cognitive functions. Our investigation entails healthy participants or those with a range of cognitive disorders, including anxiety, PTSD, and epilepsy. Furthermore, this review introduced an improvement in cognition associated with CBD interventions considering different formulations, administration doses, and routes while also exploring the role of an appropriate diet and demographic factors in enhancing CBD effects. Given the growing interest in CBD, the study aims to contribute valuable information to the relationship between CBD and cognitive function in a diverse population.

## Methods

This review followed the five steps of the Academy of Dietetics and Nutrition for conducting an evidence-based review (AND, 2022). A systematic literature search used PubMed and the resources of the Florida International University Library to identify high-quality primary reports from clinical trials using CBD as an intervention to treat neurocognitive disorders.

**FIGURE 1:**
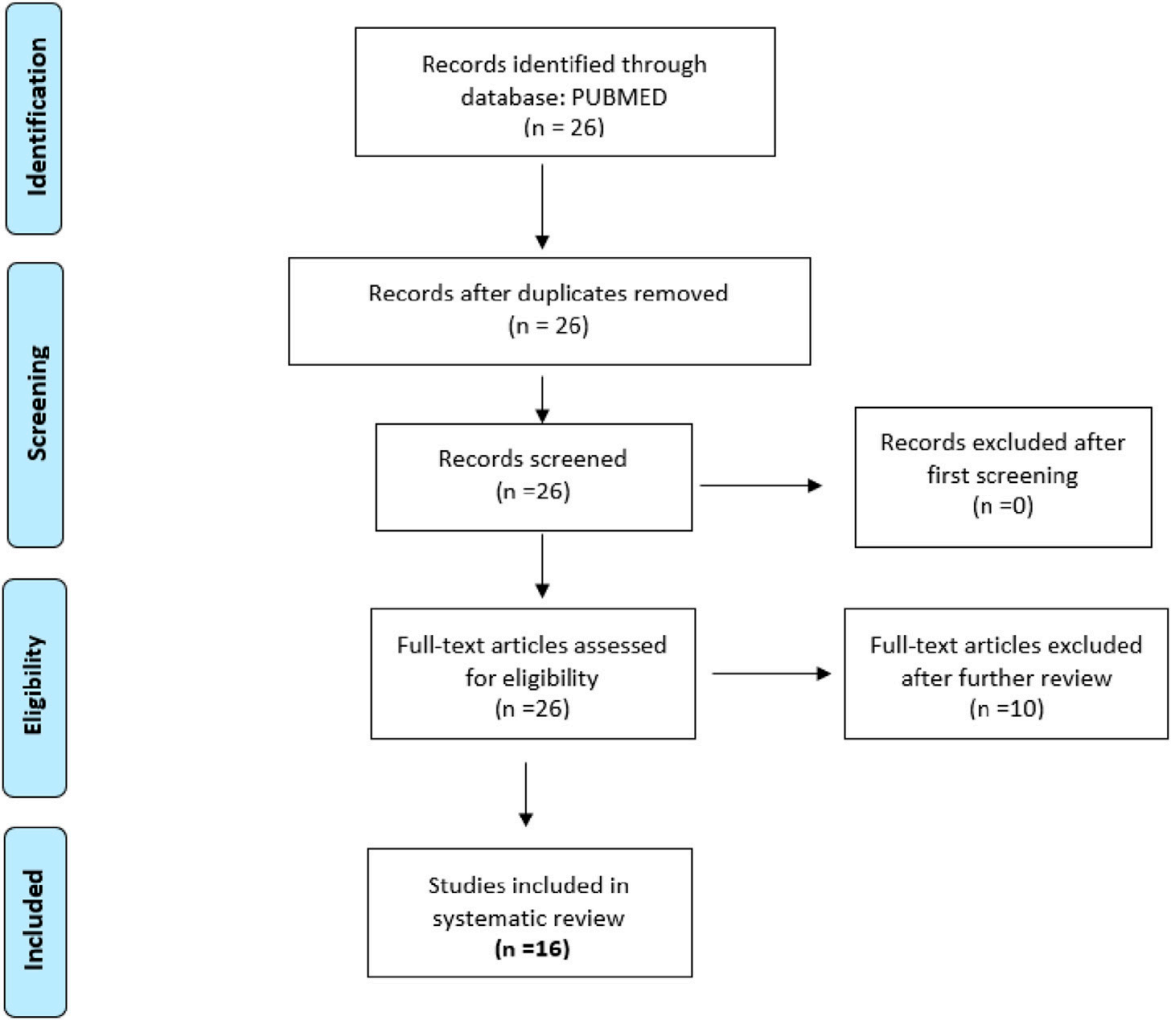
Consort flow diagram.

The inclusion criteria for articles were primary reports of randomized, controlled, and uncontrolled clinical trials that included healthy participants or participants older than 12 years with cognitive disorders, sample size with more than four individuals per group, year range from 2013 to 2023, and language limited to English and Spanish. After examination, articles that did not meet the inclusion criteria were excluded (Table 1). Two searches were conducted. The keywords used for the first search were CBD, cognitive, dementia, not pediatric, not corticobasal, not schizophrenia, and not multiple sclerosis. The filters applied were as follows: Clinical Study, Clinical Trial, Clinical Trial, Phase I, Clinical Trial, Phase II, Clinical Trial, Phase III, Clinical Trial, Phase IV, Randomized Controlled Trial, Humans, English, Spanish, Female, Male, Adult older than 19 years, and from 2013/1/1–2023/12/31. The final selection included 14 articles (Figure 1). The second search included dementia as a keyword, Cannabidiol AND Dementia NOT pediatric NOT pediatric NOT autism NOT corticobasal Filters: Clinical Trial, Clinical Trial, Phase I, Clinical Trial, Phase II, Clinical Trial, Phase III, Clinical Trial, Phase IV, Randomized Controlled Trial, in the last 10 years, Humans, English, Spanish, Female, Male, Adult: 19+ years. Articles that did not report CBD only as a treatment were excluded. The research question that guided the review was whether CBD administration could improve cognitive impairments in adults >12 years old. Graphical Abstract was created with Biorender.com/aynda001@fiu.edu/ayndarta@fiu.edu.

**TABLE 1:** The inclusion, exclusion criteria, and detailed search strategy employed.

Inclusion criteria	Exclusion criteria
Age: >12 years	Age: <12 years
Setting: Any	RCT that did not include CBD as an intervention. Meta-analysis
Health status: Any	Pregnant or nursing females Cancer or other medically significant conditions or acute systemic disorders
RCT or clinically controlled or uncontrolled studies	Observational studies Prospective and retrospective cohort studies
Size of study groups: The sample size is more than four individuals for each study group	
Year range: 2013–01–01 to 2023–12–31	Prior to 2012-12-31
Language: Limited to articles published in English and Spanish	Language: Articles not published in English or Spanish

## Results and discussion

CBD is a compound that has gained popularity due to its promising effects. Cognitive impairments are on the rise, and finding alternative treatments is imperative. This article explored using CBD as an alternative to conventional cognition treatments. Understanding and treating cognitive impairments is crucial, as one in nine adults (11%) experience a subjective cognitive decline (CDC, 2024). Additionally, anxiety disorders affect 40 million adults (19.1% of the population aged 18 and older) every year (ADAA, 2024). With this prevalence of cognitive impairments, it is essential to develop effective alternative interventions. Therefore, exploring the effects of CBD on cognition will allow us to advance our understanding of its therapeutic potential. The summary of the included trials is described in Table 2.

**TABLE 2:** Randomized clinical trials of CBD intervention to treat cognitive disorders.

Author, year	Study design	Inclusion/Exclusion	Exposure	Study outcomes
1 Berger et al. (2022) + PMID 35921510	Open-label, single-arm, randomized, uncontrolled trial	I: 12–25 years diagnosed with Anxiety by DSM-5. No improvement with CBT and/or antidepressant medication. Fluent in English and 6 weeks of stable antidepressant dosage E: Schizophrenia spectrum, delusional, and bipolar I disorder, substance or medication that induced psychotic disorder, sensitivity, or allergy to CBD, taking any medication that can interact or affect CBD metabolism, taking any antipsychotic or anxiolytic medication. Any pregnant, nursing, or an effective method of contraception, severe drug or alcohol dependence, liver or thyroid condition, or acute systemic disorder, unable to sign the consent, not a stable dose of an antidepressant for 6 weeks	Length = 12 weeks, N = 30 Increase of CBD 200 mg/week with respect to AE or no improvement in cognitive function until 800 mg Oral capsule of CBD containing 200 mg, a blend of even saturated unbranched numbered triglycerides in vegetable oil Week 1: 400 mg/day Week 4: 600 mg/day Week 8: 800 mg/day for 8 weeks Follow-up visits @ 4 weeks, 8 weeks, 12 weeks, and 26 weeks Blood withdrawal and physician visits every 4 weeks CBT every 2 weeks	ITT analysis: decreased anxiety severity 12 weeks 19 PP max dose 800 mg; nine PP max dose 600 mg; one PP 400 mg Reduced Overall Anxiety Severity and Impairment Scale at week 12. [−42.6% reduction (p < .0001)] 12 PP reduced anxiety by 50% by week 12 18 PP reduced anxiety by 33% by week 12 Anxiety decreased (HARS) −50.2% from t = 0 Depression symptom severity: 29.9% at week 12 with respect to baseline Social and occupational functioning +11.3% on SOFAS. NS decreased anxiety, depressive symptoms, and social and occupational functioning after 6 months CGI improved for 53.3% of PP by week 12. Markedly or severely ill decreased from 17 (56.7%) to five (16.7%) PP in CGI. pCBD increased at week 4 to 43.5 ng/mL (5.7%); at week 8, to 74.1 ng/mL (7.7%), and to 68.7 ng/mL (8.4%) at week 12. 600 mg/d reached the maximum pCBD, not 800 No correlation of CBD and outcome week 12 (r = −0.14, p = .46) No correlation between pCBD and a reduction in overall anxiety scores (r = −0.004, p = 0.83) 12 weeks CBD improved anxiety n PP receiving an antidepressant (−3.5; p = 0.003) or not (−5.6; p < 0.001). PP taking antidepressants had higher AE (OR = 6.4; 95% CI, 1.16–35.44; p = .03) Adverse events not pCBD-related (ρ = −0.03, p = .88) Only mild adverse events in 19/31 PP No clinically significant RBC or WBC, renal or liver function Citalopram or escitalopram increased pCBD after 12 weeks
2 Arkell et al. (2020) + PMID 33258890	Crossover, double-blind, RCT	I: Healthy 20–50 years, self-reported cannabis use < 2×/week in the last 12 months and >10 lifetime exposures, valid driver’s license with at least 2 years’ driving experience and driving more than 2,000 km/year, BMI 20–28, absence of an endocrine or neurological condition, and written informed consent E: Endocrine, liver dysfunction, or neurological condition; history of drug abuse, addiction, cardiac dysfunction, and/or current psychiatric disorder, adverse effects with previous use of cannabis, current use of medications known to affect driving, QT syndrome, active hypertension, pregnancy, or lactating	N = 22 Length: 1 month 4× Treatment Four session with at least 7–28 days in between inhaled G1: 13.75 mg THC only G2: 13.75 mg: 13.75 mg THC/CBD only G3: CBD 13.75 mg G4: placebo Driving test at 40–100 min and 240–300 min Cognitive test at 0 min, 5 min, 135 min, and 205 min and at the start of the trial Blood sample, BP, HR at baseline, 0 min, 25 min, 130 min, 200 min, and 320 min Subjective drug effects using VAS at 0 min, 25 min, 130 min, 200 min, and 240 min post vaporization	40–100 min post THC and THC/CBD compared to placebo had higher impaired driving p < .001 CBD compared to placebo did not affect impaired driving THC rated more driving impaired vs. placebo at 100 min (p < .001) and 300 min (p = .008) THC/CBD driving impaired vs. placebo (p < .001) at 100 min and (p = .001) at 300 min At 100 min, PP rated their quality of driving worse with THC (p = 0.01) and THC/CBD (p = .006) compared to placebo CBD alone failed to significantly improve cognitive or psychomotor condition or impairment vs. placebo THC/CBD caused less anxiety, reduced strength of drug effects, and greater confidence in driving vs. THC alone Lowest number of PP with driving impairments using CBD when results considered alcohol levels
3 Bloomfield et al. (2022) + PMID 35445839	RCT double-blind, placebo crossover	I: Healthy PP, English speaking, 18–70 years, low Beck anxiety inventory (BAI) score, and right-handed E: Lifetime CBD use, using psychotropic drugs, current or history of mood, psychotic, psychiatric, drug abuse, <5×/lifetime of recreational drug use other than cannabis, functional MRI contradictions, nicotine dependence, >7 scores on Alcohol Use Disorders Identification Test, pregnancy, lack of capacity sign concern, needle phobia, color blindness, allergies to cannabis, microcrystalline cellulose, gelatin or lactose, and/or unwillingness to take CBD	Length: 2 weeks 2× CBD/week, N = 24 12F/12M 600 mg of oral pure synthetic CBD Placebo Two cognitive and fMRI testing sessions, 9 days/in between. 12 opaque capsules, identical to placebo lactose cap; pCBD measured 4 h post treatment. Fast from midnight, allowed caffeine Mood and face rating task by 11- or 7-point VAS, respectively, HR, and BP taken 10 min before treatment administration and 0 h.5 h, 2 h, 4 h, and 6 h post-drug administration The mental arithmetic task recorded HR, BP, and VAS scores four times without (pre-control, post-control) and with (pre-stress, post-stress) stress conditions at 5:30 h; fMRI 2:30 h post-drug administration. Sandwich, snack, drink provided at 4 h	pCBD higher than placebo (median = 0 ng/mL, IQR = 0) CBD group experienced more anxiety than placebo (median = 6.01 ng/mL, IQR = 4.24) (pre and post) p = 0.048 from pre- to post-stress, not supported by Bayesian analysis suggesting Type 1 error NS drug effects for self-reports of stress, calm, or relaxed subjective anxiety or happiness and for HR and BP Stress tasks did not induce high scores of self-reported anxiety or stress (p = 0.018) Decreased anxiety from baseline to 1 h post drug. NS effect of time on “happy” scores and SBP. pCBD median = 6.01 ng/mL compared to 0 in placebo. CBD did not produce effects on brain responses to emotional faces and cognitive measures of emotional processing or modulate experimentally induced anxiety relative to a placebo
4 Bolsoni et al. (2022a) + PMID 35029706	Double-blind, randomized trial	I: 18–60 years, PP diagnosed with PTSD according to DSM5 criteria E: History of drug dependence or abuse, other psychiatric disorders (except depressive or anxious), and presence of organic brain syndrome	Length–3 weeks, N = 33 3× sessions at 1-week intervals 1× treatment of 300 mg CBD and determine anxiety First session: PP signed consent; recorded trauma event and imagined it for 30 s Second session: 15 min habituation, CBD 300 mg dissolved in corn oil or placebo in corn oil in a gelatin capsule given, wait 1:30 h, and measure BP, HR, SC, STAI-E, VAMS, listen to trauma event, imagine it, and repeat measures Third session, no CBD or placebo, same protocol as the second session	NS effects of CBD on anxiety, discomfort, and sedation CBD helped decrease the cognitive impairments during trauma recall and persisted over time (1.5 h p = 0.03 and 1 week p = 0.04) NS differences in SC, HR, and DBP
5 Schoedel et al. (2018) + PMID 30286443	Randomized, double-blind, controlled crossover trial	I: Healthy 18–55 years with BMI 19.0–30.0 and weight of ≥50 kg ≥10× experiences with nontherapeutic CNS depressants (opioids, stimulants, hallucinogens, dissociative anesthetics, or cannabinoids) ≥1 nontherapeutic use of any drugs of abuse/lifetime, ≥1 nontherapeutic use of a CNS depressant or cannabinoid within 12 weeks and >12 weeks polydrug experiences, and negative pregnancy test/effective method of contraception Pass qualification phase (QP) (After taking treatment. VAS Emax value of 65 pts, and placebo 40–60) and tolerate treatment E: No alcohol or drugs-of-abuse dependence or addiction [Diagnostic and Statistical Manual of Mental Disorders (DSM-IV-TR)] Current or prior treatment for substance abuse disorders, IV drugs of abuse/2 years. Any condition that may affect drug absorption, distribution, metabolism, or excretion	Length: 74 d/8 d for each CBD dose with washout periods N = 41 1× each dose CBD 750; 1,500; 4,500 mg, 8 days washout: After Rx and QP, VAS score, 8 days washout between quali and oral treatments. (7 groups) After overnight fasting 1: CBD 750 mg 2: CBD 1500 mg 3: CBD 4500 mg 4: Alprazolam 2 mg 5: Dronabinol 10 mg 6: Dronabinol 30 mg. (4, 5, 6 are positive control) 7: Placebo Follow-up 8–14 days after the last treatment Subjective assessments were done at 0.5 h, 1 h, 1.5 h, 2 h, 2.5 h, 3 h, 4 h, 5 h, 6 h, 8 h, 10 h, 12 h, and 24 h post dose Cognitive and motor tests were done at 1 h, 2 h, 3 h, 6 h 8 h, 12 h, and 24 h post dose. pCBD 24 h post treat. Cardiac and pulse monitor by telemetry up to at least 12 h post drug Cognitive measures: divided attention task, Hopkins verbal learning test-revised, and digit symbol substitution task (DSST)	CBD did not cause addiction Drug liking: Placebo and CBD neutral at all time points CBD drug liking increased when the dose increased to 1,500 mg and 4,500 mg (max at 2 hr) NS differences in drug-liking VAS for CBD 750 mg vs. placebo Drug-liking VAS for 1,500 mg CBD vs. placebo (p = 0.04) and 4,500 mg of CBD vs. placebo p = 0.002) Drug-liking VAS: three doses of CBD yielded significantly lower scores than positive controls. CBD had a higher mean of positive effects vs. placebo CBD produced a small decrease in alertness/drowsiness (1–4 h post dose) and a neutral range for both positive controls in drowsiness NS cognitive effects as measured by a divided attention task, other cognitive and motor assessments No significant effect of CBD doses compared to 10 mg dronabinol vs. placebo 30 mg of dronabinol showed cognitive issues. pCBD was measured at 6 hr for 750 mg and 1,500 mg doses, and no increase was noted in the 4,500 mg dose No serious AEs related to CBD. At 1,500 mg CBD, AST, ALA, creatine, blood creatine phosphokinase increased in 3 PP
6 Lees et al. (2023) + PMID 36598543	RCT, double-blind, parallel-group, and placebo	I: 16–60 years, Cannabis use disorder at least moderate severity (≥4 symptoms by DSM-5 symptoms), capacity to give informed consent, desire to stop using cannabis (within next month), unsuccessful attempts to quit cannabis, co-administered cannabis with tobacco, positive urine THC-COOH, negative pregnancy test for women 7 days prior to starting, contraception methods during trial and 6 weeks afterward E: 16–26 years with vital signs within normal limits, pregnant, breastfeeding, allergies to CBD, microcrystalline cellulose, or gelatin, using psychotropic or other illicit drugs, positive urine test for drugs, current or previous self-reported diagnosis of a psychotic disorder, physical health problem deemed clinically significant, or not speaking English	Length – 4 weeks with daily placebo or 400 mg and 800 mg of CBD, N = 70 Stage 1: 12 PP 4 weeks CBD treatment 2×/day at home of two gelatin capsules: microcrystalline cellulose and CBD (total doses of 200 mg, 400 mg, 800 mg) Stage 2: 70 PP; 4 weeks; 400 mg CBD, 800 mg CBD, or placebo 30-min sessions of motivational interviewing at screening, baseline, and weekly for 4 weeks Text reminders every 12 h Cognitive and assessments of cannabis use at baseline, week 4, and week 12 for follow-up	200 mg CBD was not effective No effect of CBD vs. placebo (lack of dose-by-time interaction) NS dose-by-time interaction on delayed prose recall scores Significant main effect of time, improved recall in CBD groups at week 4 vs. baseline Significant dose-by-time interaction at 800 mg CBD (0.76, 95%CIs: 0.01, 1.54) but not at 400 mg CBD (0.41, 95%CIs: −0.34, 1.25) Performance improved by 0.30 (95%CIs: 0.02, 0.58) in the 800 mg group, by 0.13 (95%CIs: −0.14, 0.42) in the 400 mg group, and by −0.08 (95%CIs: −0.35, 0.1 in the placebo group No effect of CBD compared to placebo on secondary cognitive outcomes, except backward digit span, which increased in the 800 mg CBD group (0.30, 95%CIs: 0.02, 0.58) Baseline urinary THC: COOH higher for the 400 mg CBD group than 800 mg CBD and placebo groups All groups treated with 400 mg and 800 mg of CBD reported reduced cannabis use
7 Hurd et al. (2019) + PMID 31109198	Double-blind randomized placebo-controlled trial	I: Healthy 21–65 years, DSM-IV for opioid dependence, abstinent from heroin for at least a month E: Tested positive for drugs other than nicotine, dependence on other than nicotine or heroin in the last 3 months, on methadone, buprenorphine, or another opioid antagonist, significant medical history or condition, hypersensitivity to cannabinoids, or showing acute signs of heroin withdrawal	Length—2 weeks, 1× CBD for 3 consecutive days or placebo 400 mg and 800 mg of CBD. N = 42 placebo (same composition and appearance as CBD), Oral CBD (epidiolex) 400 mg/day and 800 mg/day solution (ethanol, sucralose, strawberry flavor, and refined sesame oil) Follow-up 1 week after the last drug administration Physiological stress response, positive and negative affect, visual analog scale for anxiety, and cravings recorded on days 1, 2, and 4 Heroin craving questionnaire was taken home daily after treatment Vital signs were taken before and after treatment administration Cognitive tests were performed at baseline and day 3	3–4 h high pCBD and has a half-life of 18–32 h 25.3% HIV+ 17.8% HCV+ Craving: women 2× > men, p = 0.0476. Placebo: craving > CBD treated PP, similar % CBD doses Reduced drug cues during trial craving placebo > CBD 400 mg > CBD 800 mg Significant difference in cue craving condition in all sessions, p < 0.0001, and drug cues worked to enhance craving. Craving scores constant to neutral cues Placebo showed significantly greater craving after the drug cues vs. CBD groups (800 mg of CBD = 0.23; 400 mg of CBD = 0.44). NS craving between CBD doses The 400 mg CBD did not significantly reduce craving, but the 800 mg CBD dose did reduce craving 800 mg CBD significantly reduced craving vs. placebo at the end of the trial Sex was not associated with anxiety Anxiety: placebo > CBDs p = 0.0079 p = 0.0233 > anxiety when cueing in all groups Positive PANAS CBD 400 mg > 800 mg, p = 0.0165 Negative PANAS increased in trial p < 0.0001 NS improvement in cognitive impairment baseline vs. end Negative PANAS for the placebo > 800 mg of CBD (lowest increasing negative) but NS p = 0.06
8 McCartney et al. (2022) + PMID 35637624	RCT double-blind, crossover trial	I: Healthy 18–65 years, driver’s license for ≥1 year, and not using cannabis within 3 months E: Adverse response to cannabis, use of cannabinoid products or synthetic cannabinoids, sleep disorder, suicidal thoughts or attempts, use of anticonvulsant medications, drug addiction (including cannabis) and/or alcohol dependence, major psychiatric disorder within 12 months (except clinically-managed mild depression or anxiety), BMI >30 kg/m^2^, caffeine intake >300 mg/d, current use of medications that induce or inhibit the cytochrome (CYP) 450 enzyme system or are metabolized by CYP enzymes that are inhibited by CBD, unwillingness to do pre-trial procedures or refrain from use drug during trial, sickness to simulator experiences, pregnant or lactating	Length – 1 month with four sessions, N = 17 Practice session on driving and cognitive test prior to first session Four sessions/7.5 days avg apart Overnight fasting, standardized breakfast, then administered: placebo (MCT oil), oral CBD (15 mg, 300 mg, or 1,500 mg) in MCT oil Light standardized snack provided ∼150 min post-treatment Driving performance measured 45–75 min and 210–240 min post-treatment Cognitive functions were measured at baseline, 15–45 min, and 180–210 min post-treatment, but the psychomotor vigilance task was not completed at baseline Subjective tests, HR, and BP measured between 15–45 min, 75–95 min, 140–150 min, 180–210 min, and 240–260 min post treatment. pCBD was measured at baseline and pre- and post-drives 1 and 2	CBD 300 mg (p = 0.011) and CBD 1500 mg (p = 0.007) improved DAT vs. CBD 15 mg over the trial vs. baseline. This improvement can indirectly benefit cognitive issues Better cognitive improvement was seen for CBD-300 (–0.16 ± 0.31 vs. +1.21 ± 0.43, p = 0.011) and CBD-1500 (–0.19 ± 0.43 vs. +1.21 ± 0.43, p = 0.007) groups than in the CBD-15 group NS impairment in cognitive tasks across CBD treatments Higher VAS anxiety on placebo than CBD 300 mg (p < 0.001) or CBD 1500 mg (p = 0.033) High anxiety in CBD 15 mg vs. CBD 300 mg (p = 0.001) and CBD 1500 mg vs. CBD 15 mg (p = 0.040) Detectable levels of CBD or metabolites in all PP and placebo, so washout did not work NS driving impairment across treatments
9 Flores et al. (2023) + PMID 37375567	Double-blind, RCT	I: 18–50 years, 6 weeks of abstinence from cannabis (THC or CBD), and no chronic alcohol and/or drug use E: diagnosed cardiovascular, neurological, metabolic, or mood disorders, pregnant and/or nursing Unable to adhere for 8 weeks trial, major illness that affects workout, 24 h abstention from alcohol and 12 h from caffeine prior to each section	Length – 8 weeks, N = 48 Hemp-derived CBD 50 mg/day or 225 mg/medium-chain triglyceride (MCT) as a placebo 7-day average of steps/day, self-reported cognitive function (NIH PROMIS) Cognitive Function–Abilities—Short Form 8a and Objective Function Short Form 8 (7-point Likert scale) 24-h diet recall before blood collection. Body composition, fitness, physical activity, CRP. Eight visits in total, four pre- and four post-interventions	NS differences between pre- and post-intervention cognitive scores CBD did not improve aerobic and anaerobic fitness, physical activity, mental health and wellbeing, and inflammation measures (CRP, IL1, TNF, and IL6) or myoglobulin, Ck, Lps, and claudin 3 CBD appeared to prevent reductions in peak anaerobic output (production of energy ATP) in physically active adults
10 Grimm et al. (2018) PMID 29887287	RCT, double-blind, placebo, crossover trial	I: Healthy male volunteers E: Psychiatric, other medical disease, taking medication (except stable thyroid replacement therapy), and positive drug urine test or regular drug use	Length–3 weeks with three sessions. N = 16 Standardized sandwich ∼350 kcal before single dose (capsule) Groups: placebo (saline), THC 10 mg, CBD 600 mg. (Capsule) fMRI 75 min after treatment DSS, STAI, and PANSS 120 min after treatment intake Blood was drawn before treatment and 60 min and 200 min after treatment	CBD led to an increase in frontostriatal connectivity between the putamen and prefrontal cortex compared to THC and placebo NS subjective effects on anxiety, positive and negative affect, subjective valence, and arousal ratings of THC vs. placebo or CBD vs. placebo THC had a significantly higher anxiety rate (p = 0.03) pCBD was measured 208 min after drug admin and was 0 at baseline Low pTHC during fMRI
11 Bolsoni et al. (2022b) + PMID 35293520	RCT, placebo, double-blind trial	I: 18–60 years diagnosed with PTSD by DSM-V E: Abuse or dependence on psychoactive drugs, other psychiatric disorders (except depression and anxiety disorders), neurocognitive disorder	Length – 2 weeks, N = 33 First session: signed consent and recorded traumatic event Second session (1 week later): 15 min habituation, CBD 300 mg dissolved in corn oil or placebo in corn oil in a gelatin capsule, wait 90 min for recall procedure After drug intake and before and after the recall event, BP, HR, salivary cortisol, and VAMS were measured PTSD trauma was divided into sexual (seven CBD, seven placebo) or nonsexual (ten CBD, nine placebo) Diagnosed PSTD by DSM-V and severity by the Posttraumatic Stress Disorder Checklist (PCL-5)	Baseline: significantly higher anxiety (VAMS scores) after recalling trauma Higher cognitive impairment in the placebo group than in the CBD group (p = 0.001) Higher anxiety in the placebo group than in the CBD group. CBD significantly decreased anxiety in the nonsexual trauma group compared to the sexual group (p = 0.035) Anxiety in the nonsexual trauma group before and after was lowered in the CBD group vs. the placebo group (p = 0.033), but the change was NS in sexual trauma PP Cognitive impairment was significantly lowered after CBD treatment in the nonsexual trauma PP vs. placebo (p = 0.008), but the change was NS in sexual trauma PP NS difference in sedation or discomfort in sexual vs. nonsexual trauma PP Higher SBP (p = 0.008) and HR (p = 0.04) after recall of traumatic event in both treatment groups
12 Haney et al. (2016) + PMID 26708108	Multi-site, randomized, double-blind, within-subject trial	I = 18–50-year-old healthy PP (evaluated by physical examination, psychiatric screening, electrocardiogram, BP, HR, drug screening, and adequate medical history), ½ cannabis smoked >4×/week, no other type of drug use, nicotine, or caffeine, not seeking treatment for cannabis use E = Any woman who was pregnant, nursing, or not using effective contraceptive methods, use of over-the-counter or prescribed medication, and any mental disorder condition that would benefit from medical intervention	Length – 8 weeks with one session/week, N = 31 8 sections of 8-h/week for 8 weeks 1 section/week No eating, smoking cannabis, or consuming alcohol after 12 a.m. before trial A light breakfast was provided (bagel or cereal, juice, coffee), and after 30 min, all PP received CBD capsules (200 mg, 400 mg, 800 mg) or a placebo 90 min later, ½ cannabis cigarettes smoked (puff controlled) After afternoon, self-administered cannabis cigarettes and no controlled puff After breakfast, baseline CVD tests, subjective effects tests, and cognitive battery tests were completed (15–120 min) Cannabis smoked containing inactive cannabis (0.01% THC) or active cannabis (5.30%–5.80% THC) 60 min after lunch Subjective questionnaires and performance tasks were completed at baseline and at 15–120-min intervals after capsule and cannabis administration HR and BP were measured at baseline, 30 min, 60 min, and 85 min after capsule administration and 15–150 min after cannabis administration Additional sessions with eight participants: after breakfast, 800 mg of CBD was given, and blood was drawn at baseline, 60 min, 120 min, 180 min, 240 min, 300 min, and 360 min after CBD administration	Increased ratings of “high” and “good drug effects,” liking, strength, desire to take again, and good effect with 5.30%–5.80% of THC vs. 0.01% of eTHC. p < 0.001. CBD vs. placebo did not have significant differences with respect to high” and “good drug effects.” CBD vs. placebo had NS effect on capsule ratings for high and good drug effect NS effect of CBD vs. placebo on task performance NS effects of CBD vs. placebo with active or inactive cannabinoid for cognitive tests (DSST and CPT) p < 0.01 interest in choosing to self-administer active cannabis vs. placebo cannabis for those taking CBD. Placebo with active cannabis increased HR (p < 0.01) NS increase in BP with CBD and cannabis CBD prior to cannabis did not show significance in the subjective, reinforcing, or cardiovascular effects Maximum pCBD peak concentrations (77.9 ng/ml) was obtained after 120 min of 800 mg intake of CBD. CBD did not reduce the reinforcing or positive subjective effects of cannabis in current cannabis smokers
13 Rizkallah et al. (2022) + PMID 35367279	Randomized, double-blind, controlled trial	I: 18–65 years, current cocaine user, diagnosed with cocaine use disorder E: immunodeficiency, super sensitive to cannabinoids, severe or unstable medical or psychiatric condition, other substance use disorder (except cocaine and nicotine), severe or needed pharmacological treatment, immunodeficiency, hypersensitivity to cannabinoids, unstable medical or psychiatric condition (history of schizophrenia, schizoaffective disorder, bipolar disorder, current acute psychosis, severe suicidality, or requiring pharmacological treatment during the study)	Length: 92 days, N = 50 800 mg of daily oral CBD or placebo for 92 days The cognitive assessment was measured at baseline, day 7 (phase 1), and at week 6 12-week outpatient follow-up Neuropsychological test automated battery on day 1, day 7, and at week 6. (stop signal task; SST), risky decision making (Cambridge gambling task; CGT) and visual memory (pattern recognition memory; PRM)	CBD was not effective at improving cognitive function when compared to placebo NS difference in PRM, SST, and CGT between placebo and CBD. Results were controlled for sex, severity of dependence, and baseline cognitive scores
14 Birnbaum et al. (2019) + PMID 31247132	RCT, double-blind, placebo-controlled, open-label, crossover	I: 27–79 years, localized intractable epilepsy confirmed by electroencephalography and recurrence of >4 seizures/month, patient of the MINCEP Clinic for ≥6 months, provide informed consent, be eligible to receive CBD as part of clinical care E: a recent History of epilepsy, women not practicing contraception	Length—4 weeks, N = 8 m PP fasted >10 h before. Two sessions (fasting or feeding) with 2 weeks in between Session 1 = Feeding stage: soft gelatin capsule of CBD (dissolved in coconut oil) 200 mg (n = 1) and 300 mg (n = 7), followed by 240 mL of H_2_0 and, within 30 min, consume 840–860 calories (500–600 calories from fat) of a breakfast burrito Session 2 = Fasting stage: 200 mg (n = 1) and 300 mg (n = 7) CBD, followed by H_2_0, and breakfast supplied 4 h post dose After session 2, PP took 300 mg CBD daily >2 weeks with usual food intake Blood taken at baseline, 0.5 h, 1 h, 2 h, 2.5 h, 3.5 h, 4 h, 5 h, 6 h, 24 h, 48 h, and 72-h post-treatment Battery test before and 2.5 h post treatment Seizures and adverse events were measured daily	NS (p > 0.25) difference in cognitive test scores NS change in cognitive test scores (p = 0.15) between fasting and fed NS AE NS changed in seizures Frequency in seven PP Two PP decreased seizures in the fed stage (one decreased from an average of seven to one seizure per day) and one increased during the fed state) Higher pCBD in the fed stage (126 ng/mL) than fasting (9 ng/mL) Cmax 14× in fed > fasting (p = 0.025) AUC4x fed > fasting (p = 0.008) Tmax was variable for both nutritional stages, but Tmax was reached earlier in the fed stage (2.4 h) vs. fasting (3.2 h) Half-life fasting (38.9) > fed (24.3)
15 McGuire et al. (2018); 2018 + PMID 29241357	RCT, double-blind, multicenter, placebo-controlled trial	Safety and effectiveness of CBD in patients with schizophrenia I: 18–65 years; schizophrenia or a psychotic disorder utilizing DSM-IV. E: Positive and negative syndrome score <60 at screening, presence of dementia, delirium, or similar disorder/clinical finding that could put the patient at risk, and taking more than one antipsychotic medication. Any woman who was pregnant, nursing, or not using birth control. Psychosis induced by drug abuse	N = 86 Length – 8 weeks CBD (N = 42) vs. placebo (N = 44) 1,000 mg/day of CBD for 8 weeks 10 mL of a 100 mg/mL CBD oral solution/2×/day (morning and evening) Matching placebo alongside psychotic medication Symptom severity, level of functioning, and cognitive performance by PANSS, SANS, improvement of CGI-I, Global assessment of functioning scale (GAF), and brief assessment of cognition in schizophrenia (BACS) questionnaire Functioning and sleep severity scale, body weight, waist measure, BMI, and HDL cholesterol levels Safety and tolerability of CBD.	CBD decreased positive psychotic symptoms and improved cognitive performance and level of functioning (83.4%–54.8%) vs. placebo (79.6%–63.6%) GAF treatment difference = −0.3, 95% CI = −0.5, 0.0; p = 0.044 BACS treatment difference = 1.31, 95% CI = −0.10, 2.72 No changes in inflammatory markers Strength: CBD measured at baseline and end of treatment by blood tests Limitation – Some PP tested positive for THC, affecting the interpretation of CBD effectiveness. Substance use (cannabis, alcohol, etc.) was not an exclusion criterion Self-reported No drugs or alcohol record data. No mention of medication; only White participants, and all PP were overweight. No diet recalls
16 Boggs et al. (2018) + PMID 29619533	RCT, placebo-controlled	Assess cognitive, symptomatic, and side effects of CBD in patients with chronic schizophrenia I: 18–65 years with a DSM-IV-TR diagnosis of schizophrenia and 3 months of stable treatment and no dosage change in 4 weeks. Scored ≤1 SD below the mean for the general population on the Hopkins Verbal Learning Test (HVLT) E: Past or current DSM-IV-TR diagnosis that required pharmacological treatment, substance abuse in the last 3 months, or dependence in the last 6 months. Any woman who was pregnant, nursing, or not using birth control. Currently enrolled in a weight loss program, recent exposure to the HVLT, and undergoing treatment with cognitive enhancers and/or clozapine	N = 39; Length: 6 weeks Placebo n = 18 vs. CBD group n = 18 300 mg of CBD 2×/day (total 600 mg/day) + antipsychotic medication Cognitive assessment at baseline and week 6 by MATRICS consensus cognitive battery (MCCB) PANSS at baseline, biweekly Side effects are measured by the Barnes akathisia scale (BAS), the Simpson Angus scale (SAS), the abnormal involuntary movements scale (AIMS), and the UKU side effect scale	NS CBD vs. placebo in treating cognitive impairments No adverse events No worsening of psychosis, mood, or suicidality Sedation > in CBD vs. placebo Self-report adherences 80% power Significant drug × time effect (F (1, 32) = 5.94; p = 0.02) Reasoning and problem solving (MCCB domain) main effect of time (F (1, 33) = 3.48; p = 0.07) and a drug × time interaction (F (1, 33) = 4.47; p = 0.04) Limitations: self-report, CBD dosage used for chronically ill, intervention, and utilizing medication only

After closely analyzing all the results, CBD demonstrated improvement in anxiety and cognitive impairments in nine of 16 trials (Arkell et al., 2020; Berger et al., 2022; Bolsoni et al., 2022a; Bolsoni et al., 2022b; Grimm et al., 2018; Hurd et al., 2019; Lees et al., 2023; McCartney et al., 2022; McGuire et al., 2018). This limited consistency can be explained by various factors, including diversity of the assayed population, variability of trial designs, small sample size, underpowered studies, and uncontrolled results for comorbidities, medications, severity of dependence on drugs, etc. In addition, the variable fragile compliance and adherence monitoring in trials such as pill counting, checking the empty bottle, controlling puffs, plasma CBD, breathalyzer, daily records, different questionnaires, self-reporting, purity of formulation, and wide ranges of doses (200–1,500 mg) can account further for this inconsistency. Importantly, the CBD dose, formulation, method of administration, length of CBD treatment, and participants’ demographic characteristics and habits, such as BMI, age, race, food consumption pattern, hydration, physical activity, and more, may also account for the variability of results.

For instance, Berger et al. (2022) conducted a 12-week study intervention with a high-purity CBD combined with cognitive therapies. This trial investigated the effect of a dose escalation protocol from 200 mg/day to 800 mg/day of oral CBD on cognition. Thirty young participants with anxiety disorders who had previously failed to improve using standard treatments were included. CBD reduced the severity of the anxiety scale by 42.6% at week 12. In 12 participants, the reduction was 50%, while in 18 participants, the reduction was 33%. Additionally, depression symptom severity decreased by 29.9%, and social and occupational functioning increased by 11.3%. An enhancement of clinical global impression was observed at week 12 with respect to baseline in treated participants. These findings were not reproduced at the 6-month follow-up. To ensure treatment adherence, both plasma CBD and pill count were measured. The intention to treat analysis showed a potential decrease in the severity of anxiety at 12 weeks. CBD inhibits the cytochrome P450, which is an enzyme used to metabolize antidepressant drugs. In fact, participants taking antidepressants showed more adverse events (OR = 6.4; 95% CI, 1.16–35.44; p = 0.03) unrelated to plasma CBD (Spearman ρ = −0.03, p = 0.088) that were possibly produced by interferences of cannabidiol with antidepressant drug metabolization. Cannabidiol had an acceptable safety profile with no clinical changes in blood parameters. Several limitations were observed in this trial, such as the small sample size, which was not determined by power analysis, and results were not stratified per gender, education, marital status, or antidepressant drug (Nagendrababu et al., 2021). Moreover, the unblinded trial may have produced expectancy bias due to CBD marketing benefits and the fact that uncontrolled trial design prevents the determination of cause and effect. Dietary information, BMI, and recreational drugs were not recorded, which could influence the presented results (Chayasirisobhon, 2020; Robbins, 2011; Birnbaum et al., 2019).

Similarly, Bloomfield et al. (2022) conducted an RCT crossover study to investigate the behavioral and neural effects of 600 mg of oral CBD in 24 healthy participants. The study reported that CBD had an effect in increasing anxiety when pre- and post-treatment were assessed in the treated group. However, CBD had no effect on a range of emotional measures relative to placebo. There were no other differences between groups for anxiety, cognition, and physiological measures. Several limitations must be considered when interpreting these results. The study used two different surveys to measure anxiety at baseline and post-treatment; these surveys introduced variability into the trial, and two different sets of emotional faces were used to test the same outcome. Participants self-reported fasting conditions prior to the session, but no 24-h dietary recall was conducted. Participants were provided with a standardized meal and drinks. Furthermore, the capsules were not oil-based, and neuroimaging results were not analyzed with respect to groups. On a positive note, treatment and placebo groups were randomized and balanced for sex. Power was analyzed based on CBD acute effects, and participants underwent drug screening, breathalyzer tests, and pregnancy tests, if applicable.

Arkell et al. (2020) studied the effect of THC and CBD on driving as a model to measure cognition impairments in 22 healthy participants with previous cannabis experience. The trial was performed in four sessions within a month. Participants inhaled 13.75 mg of THC and/or CDB vaporized doses. Results showed that driving impairments were significant at 40–100 min (p < 0.001) post vaporization of THC and CBD in combination with THC. The CBD did not impair driving compared to placebo, while THC rated more impaired driving at 100 (p < 0.001) and 300 min (p = 0.008) than the placebo. Additionally, the group that combined THC/CBD showed higher driving impairment vs. the placebo at 100 min (p = 0.001) and 300 min, suggesting that the THC had predominantly impairment effects on cognition. When results were controlled for alcohol levels, CBD showed decreased driving deficiencies. This trial was not powered by the detection of CBD on driving, and dietary information was not recorded. Participants self-reported taking painkillers and/or medications that could have altered treatment metabolism. The dose used for CBD was lower than that used in previous trials. In the trial, participants used a breathalyzer for alcohol and a drug screener in urine tests at the beginning of a session, and standardized procedures to inhale drugs, drive, rest, and lunch were performed. The trial reported good adherence powered by inside treatments.

In a different study, McCartney et al. (2022) investigated the effects of CBD on driving performance over four sessions in 1 month involving 17 healthy participants. The placebo and CBD doses of 15 mg, 300 mg, and 1,500 mg were taken with a standardized breakfast and a light snack. Compared to the baseline, CBD 300 mg (p = 0.011) and CBD 1,500 mg (p = 0.007) improved in the divided attention task at the end of the trial. No other significant impairments or improvements were observed in cognitive and driving tasks across the CBD doses. Participants reported higher levels of anxiety on the placebo than on CBD doses. However, several limitations should be considered. The study required a 24-h diet recall, but the results were neither presented nor analyzed. The driving performance was measured on a stimulator, which may not fully replicate real-world conditions. The trial was underpowered, and the washout period between treatments was not sufficient to avoid carry-over effects, as detectable levels of CBD or metabolites were found in all participants. On the other hand, prior to sessions, breathalyzer, urine, and dehydration tests were performed, and participants had a standardized breakfast and snack. In addition, the exact formulation of CBD in medium-chain triglyceride oil was used throughout the study and participants and had no serious adverse events.

In an attempt to clarify the differential effects of the most popular types of cannabinoids in healthy populations, Grimm et al. (2018) investigated whether CBD or THC-enhanced neuronal pathways are associated with neuropsychiatric conditions and cognition improvement. Sixteen healthy participants took a single dose of placebo, or 10 mg THC, or 600 mg CBD. CBD increased frontostriatal connectivity compared to THC and placebo. This brain region is involved in learning, language, reward, motor, and addiction. However, the increase in brain connectivity did not correlate with changes in cognitive tests, and there were no significant effects on anxiety, positive and negative effect, dissociative symptom scales, and subjective valence and arousal ratings for THC vs. placebo or CBD vs. placebo. Some limitations should be considered. This trial did not show demographics, BMI, comorbidities, or baseline characteristics with respect to anxiety and perception related to cognition, and neither did the results stratify this variance. Additionally, results and symptoms were self-reported, and power was based on within-subject, with no information presented about whether it reached 80%. All participants were men, and no information on CBD purity, brand, or carrier vehicle was reported. Furthermore, the trial did not report the possible consumption or experience with THC, CBD, or any illicit drug, and different doses were used for both cannabinoids. On a positive note, participants had their plasma CBD and THC levels measured throughout the study, and participants were excluded from analysis if no detectable plasma concentrations were found. Participants received standardized meals prior to treatment.

Haney et al. (2016) obtained different outcomes. Haney et al. (2016) assessed the subjective, cognitive, reinforcing, and physiological effects of consuming CBD prior to smoking cannabis. The study included 31 participants, and 200 mg, 400 mg, and 800 mg of CBD were provided before participants smoked a cannabis cigarette with inactive and active concentrations of THC. CBD did not show significance in the subjective, reinforcing, or cardiovascular effects and did not reduce the reinforcing or positive subjective effects of the two active cannabis concentrations used. Throughout the study, participants were allowed to smoke nicotine and purchase three additional cannabis puffs, and CBD was well-tolerated and produced no significant psychoactive or cardiovascular effects relative to the placebo. However, 24-h dietary recall, power calculation, and dose effects were not recorded. In addition, plasma CBD was only measured in the last session.

Partial benefits were found in the first trial conducted by Bolsoni et al. (2022a); however, the findings changed when results were stratified by type of trauma (Bolsoni et al., 2022b). Bolsoni et al. (2022a) found that CBD had no significant effects on anxiety, discomfort, sedation, salivary cortisol, heart rate, and diastolic blood pressure. In this trial, a single dose of 300 mg of high-purity CBD or a placebo was administered to 33 patients suffering from post-traumatic stress disorder. CBD helped decrease cognitive impairment associated with trauma recall, and this effect persisted over time (1.5 h p = 0.03 and 1 week p = 0.04 post treatment). This study has several limitations to consider. Treatment was administered only once. The study did not include a 24-h dietary recall, drug screening, or other covariates that could potentially affect the efficacy of CBD. Moreover, the study had a small sample size and did not stratify CBD results based on participants’ comorbidities, but groups were matched by sex, age, BMI, and severity of PTSD symptoms. In a separate article, Bolsoni et al. (2022b) assessed whether CBD attenuates anxiety in 33 patients with post-traumatic stress disorder in a 2-week study where CBD 300 mg or placebo was administrated once. PTSD trauma was divided into sexual and nonsexual trauma. CBD lowered anxiety in nonsexual (p = 0.035) vs. sexual trauma. Additionally, CBD decreased anxiety related to nonsexual trauma (p = 0.033) and cognitive impairment (p = 0.008) after treatment compared to placebo. It is important to consider that participants in the sexual trauma group were younger; therefore, the traumatic event occurred more recently. Participants had no urine or breathalyzer test before treatment. Furthermore, comorbidities were not specified, and neither 24-h dietary recall nor medications that could impact CBD metabolism were recorded. On the other hand, groups were randomized by sex, age, BMI, and trauma severity, and CBD had a high purity.

Despite the positive findings mentioned in previous trials with CBD, Flores et al. (2023) and Birnbaum et al. (2019) did not find a significant enhancement in cognition or inflammation. Flores et al. (2023) assessed the effectiveness of CBD on aerobic and anerobic fitness, physical activity, mental health and wellbeing, and inflammation in 48 healthy participants. It was an 8-week study where participants consumed 50 mg of CBD or 225 mg of MCT oil daily. CBD did not improve cognitive function, well-being aspect, or inflammation markers (C-reactive protein (CRP), interleukin-1 and 6, tumor necrosis factor, claudin 3, and myoglobulin). The study reported relaxed methods of compliance. The authors did not refer to how consumption of CBD was tracked, recorded daily, or remembered. Moreover, no blood or urine CBD or THC tests were performed, and the calculated power of the study was based on the reduction of CRP in active adults. Dietary recall was not recorded, and surveys for cognitive function did not target attention, working memory, and executive functions.

Birnbaum et al. (2019) evaluated the kinetics of CBD administered with and without a high-fat meal in eight adults with refractory epilepsy. CBD was administered in the fed stage with a high-fat breakfast and in the fasting stage with water. Higher plasma CBD levels were seen in the fed stage (126 ng/mL fed vs. 9 ng/mL fasting). There were no significant changes in cognitive test scores, adverse events, or changes in seizures between the fasting and feeding stages assessed using self-reports. The study reached 80% power, but the results were not stratified. No 24-h dietary recall was collected, and more studies are required to determine drug efficacy vs. food.

In addition to considering the drug’s efficacy, it is also important to investigate whether a drug has addictive properties. Schoedel et al. (2018) conducted a study to investigate the abuse potential of high-purity CBD in 41 healthy recreational polydrug users in a crossover trial. Participants were administered different CBD doses of 750 mg, 1,500 mg, and 4,500 mg while fasting. The study reported that CBD did not lead to drug addiction or adverse events, and all CBD doses demonstrated significantly lower drug-liking visual analog scale (VAS) scores than positive controls. Furthermore, CBD had no significant effect on cognitive or motor assessments compared to placebo. Several limitations should be considered. Most participants were White and men, limiting the generalizability of the results. The use of subjective measures, such as VAS, might introduce bias. The study relied on self-reporting, and no drug screening or dietary recall was recorded. CBD was administered during fasting and in a single dose, which may not reflect real-life conditions and results. Additionally, the study results were not stratified to account for the use of depressants, opioids, and derivatives among the polydrugs used. On a positive note, the study had a positive control group that served to validate the experimental procedure, and all drugs were administered within the testing center. Furthermore, the study had more than 80% power based on a 15-point difference in Emax scores on a drug-liking VAS of CBD and anxiolytic drugs.

Based on these results and the presence of cognitive issues in the drug-addicted population, Lees et al. (2023), Hurd et al. (2019), and Rizkallah et al. (2022) conducted clinical trials to investigate whether CBD could diminish the craving for illicit drugs and cognition problems. Lees et al. (2023) investigated whether CBD attenuates cognitive disorders produced by cannabis use disorders (CUD) in 70 participants over a 4-week study. Placebo and CBD doses of 400 mg and 800 mg were administered twice a day throughout the length of the study. CBD demonstrated improvement in the recall task at week 4 compared to baseline, and performance in the backward digit span, a type of cognitive test, increased in the 800 mg of CBD-treated group. However, no significant effects of CBD were observed on other secondary cognitive outcomes compared to placebo. At the end of the study, all participants reported reduced cannabis use. The treatment adherence was measured by diary cards and the return of the pill box, with urine tests performed at baseline, week 4, and week 12. The study was divided into two stages: stage 1 was dedicated to investigating which CBD dose was most effective in reducing cannabis use, and stage 2 further expanded on the effects of the chosen effective doses. Limitations such as instructed time of day to ingest capsules, dietary information, and BMI were not recorded. In addition, due to continued exposure to the cognitive tasks and environment through the trial, participants might have experienced lower anxiety and scored higher. In the same way, Hurd et al. (2019) investigated the potential short and acute effects of CBD in reducing cue-induced craving and anxiety in 42 heroin patients. Participants took the placebo or Epidiolex containing CBD 400 mg or 800 mg for 3 consecutive days. After assessing patients, no significant changes in cognitive performance between baseline and end of study were found. Both CBD dosages were effective in reducing cue-induced cravings for heroin and anxiety, and the 800 mg/d dosage showed the best outcome. This study has several limitations, such as no power calculation and lack of dietary recall, and not all participants satisfied the inclusion criterion of abstaining from heroin for at least 1 month. Furthermore, no drug abuse test was conducted during the trial after the initial test at the beginning, and results were not extrapolated for participants’ conditions, such as HIV and hepatitis C virus, which could affect the interpretation of results. Craving and anxiety outcomes were subjectively measured. The sample size was small, with high diversity in the BMI ranges and mostly male participants. Lastly, Rizkallah et al. (2022) tested whether CBD was effective at improving cognitive function in individuals with cocaine use disorder in a 3-month study. Participants took oral CBD 800 mg or placebo daily for 92 days. CBD was not effective at improving cognitive function, and there were no significant differences in pattern recognition memory, stop signal task, and Cambridge gambling task compared to placebo. Participants were excluded from the study if they had additional substance use disorders. The study had no control group, power calculation, or 24-h recall recorded. Additionally, participants’ compliance and treatment adherence were not measured or recorded, and the trial had high attrition rates of 28% in Phase I and 22% in Phase II.

## Implications and outcomes of the studies for future work

A systematic review and meta-analysis using Bayesian analysis on preclinical studies found that preexisting anxiety conditions in animals predicted more significant effects of CBD than on unconditioned anxiety (Kwee et al., 2023). Likewise, Berger et al. (2022) obtained satisfactory outcomes for the reduction of anxiety and cognitive improvement by CBD when treating patients with preexisting anxiety and resistance compared to standard-of-care treatments. Bolsoni et al. (2022b) conducted a secondary analysis from a previous trial (Bolsoni et al., 2022a) and elucidated that CBD not only improved cognition but also protected against anxiety when a nonsexual trauma was established and recalled (Bolsoni et al., 2022b). In contrast to previous results, Bloomfield et al. (2022) did not obtain significant results on cognitive measures or modulation of experimentally induced anxiety by CBD in healthy patients with low anxiety levels.

Cannabis use disorders and associated cognitive issues led us to four studies that met our search criteria. Among them, Arkell et al. (2020), McCartney et al. (2022), and Lees et al. (2023) reported improvement in cognition functions, while Flores et al. (2023) did not. This divergence in findings underscores the complexity of the topic and the need for further research. Note that doses of more than 300 mg for oral administration consistently led to better outcomes. Flores et al. (2023) used doses as low as 50 mg/day for 8 weeks, while McCartney et al. (2022) and Lees et al. (2023) achieved expected results with doses of more than 300 mg once a week or twice daily, respectively. Interestingly, Arkell et al. (2020) obtained their results using only 13.75 mg/week primarily through an inhaled preparation that offered a higher percentage of CBD availability and rapid distribution to the brain with no reported serious adverse events.

Respecting other types of non-cannabinoid drugs, such as opioids and cocaine, three trials (Hurd et al., 2019; Schoedel et al., 2018; Rizkallah et al., 2022) explored the use of CBD as an anxiolytic, cognitive improvement agent, and for the reduction of drug craving (Hurd et al., 2019). None of them showed the expected results for cognition (Hurd et al., 2019), even when they included variable oral doses such as 750/1500/4,500 mg as single doses (Schoedel et al., 2018), 400/800 mg for three consecutive days, or 800 mg/day/14weeks (Rizkallah et al., 2022). At the same time, the anxiolytic action of CBD was achieved in opioid users, while Rizkallah et al. (2022) did not assess anxiety in cocaine-user participants. CBD reduced drug cue cravings in opioid users. Results in this work were not extrapolated to the type of abused drugs that affect our analysis for generalization.

Grimm et al. (2018) was the only included study that used healthy human subjects and reported an increase of frontostriatal connectivity as an indirect measure of cognition improvement in healthy individuals led by CBD treatment.

McGuire et al. (2018) and Boggs et al. (2018) studied the effect of CBD on the improvement of cognitive issues associated with schizophrenia and obtained different results. McGuire et al. (2018) found that CBD decreased positive psychotic symptoms and improved cognitive performance and level of functioning after the administration of 1,000 mg/day of oral CBD for 8 weeks in adults aged 18–65 with schizophrenia. These findings are in contrast to the outcomes reported by Boggs et al. (2018), who gave 600 mg of CBD daily for 6 weeks without interrupting the antipsychotic medication. The differences in findings could be influenced by variable doses used, sample size, length of the trials, and the masking effects of drugs of abuse included in the outcomes of McGuire et al. (2018) and not in the Boggs et al. (2018) trial. In addition, both teams assessed self-report adherences; one of them used overweight participants, and the results were not extrapolated to the type of medication used. It is important to highlight that even in schizophrenia patients, CBD showed a safe and tolerable profile, with no adverse events and no worsening of psychosis or mood or suicidality.

## Conclusion and prospects

Overall, oral doses of 300 mg–1,500 mg or unique vaporized 13.75 mg of CBD in different administration schemes were effective at treating anxiety and cognition (Arkell et al., 2020; Berger et al., 2022; Bolsoni et al., 2022a; Bolsoni et al., 2022b; Grimm et al., 2018; Hurd et al., 2019; Lees et al., 2023; McCartney et al., 2022; McGuire et al., 2018). The improvements were seen in participants with post-traumatic stress disorders, anxiety, drug of abuse (heroin, cannabis), schizophrenia, and healthy subjects. In contrast, seven trial results did not show a significant effect on treating cognitive impairments, which can be explained by various factors such as the presence of THC or other abuse drugs that can interfere with the CBD effect, low dose, and unknown purity of CBD (Birnbaum et al., 2019; Bloomfield et al., 2022; Haney et al., 2016; Flores et al., 2023; Schoedel et al., 2018; Rizkallah et al., 2022; Boggs et al., 2018). Overall, CBD showed a safe profile with scarce or no serious adverse events and no drug addiction behavior (Arkell et al., 2020; Berger et al., 2022; Bolsoni et al., 2022a; Grimm et al., 2018; Hurd et al., 2019; Lees et al., 2023; McCartney et al., 2022; McGuire et al., 2018; Birnbaum et al., 2019; Bloomfield et al., 2022; Haney et al., 2016; Flores et al., 2023; Schoedel et al., 2018; Rizkallah et al., 2022; Boggs et al., 2018). Furthermore, CBD shows potential properties that can be tested in Alzheimer’s disease treatment, and no reported clinical trial uses CBD as a primary intervention for the improvement of cognitive disorders associated with Alzheimer’s disease.

Despite CBD’s established safety profile, a significant need for more investigation remains. This includes elucidating the optimal dose, formulation, treatment length, effective administration route, and associated diet for achieving cognitive improvement through CBD treatment. The potential of CBD in Alzheimer’s disease treatment is promising, but it requires the active involvement of the medical community and researchers. Notably, no clinical trial has yet utilized CBD as a primary intervention for Alzheimer’s disease improvement.

In summary, although many of the studies support the notion that CBD is a promising compound for treating cognitive disorders, there is insufficient evidence to safely conclude that CBD is a good candidate for this purpose. Of 16 studied trials, nine significantly showed improvement in cognitive issues over a range of CBD doses. However, the absence of a general pattern makes it difficult to generate a common recommendation, and more trials are needed for conclusive evidence of CBD’s beneficial role on cognition.

This review may help summarize published work related to CBD as a candidate for cognitive dysfunction and anxiety treatment in healthy and non-healthy participants, inclusively to reduce craving for addictive drugs. Furthermore, it can open avenues for new trial designs and applications in other neurodegenerative diseases, such as Alzheimer’s disease and Parkinson’s disease, as a research priority.

## Abbreviations

AIMS, abnormal involuntary movements scale; AE, adverse events; AUC, Area under the curve; BACS, brief assessment of cognition in schizophrenia; BAS, Barnes akathisia scale; BMI, body mass index; BP, blood pressure; CBT, cognitive behavioral therapy; CGI, clinical global impressions scale; CLTS, controls; DAT, divided attention task; DBP, diastolic blood pressure; DSS, dissociative symptoms scale; GAF, global assessment of functioning scale; HR, heart rate; ITT analysis, intention to treat analysis; MCT, medium-chain triglyceride; PANAS, positive and negative affect schedule; PANSS, positive and negative symptom scale; pCBD plasma CBD concentration; PP, participants; PTSD, post-traumatic stress disorders; RBC, red blood cells; SAS, Simpson Angus scale; SBP, systolic blood pressure; SC, salivary cortisol; STAI, state anxiety inventory; VAMS, visual analog mood scale; WBC, white blood cells.
